# Potential of IL-33 as a Novel Myokine in Sarcopenia-Osteoporosis Communication: Insights from Mendelian Randomization

**DOI:** 10.2174/0118715303385833250826052804

**Published:** 2026-07-21

**Authors:** Mingchong Liu, Jiaming Wang, Xiao Fu, Chensong Yang, Jinglei Zhao, Guixin Sun

**Affiliations:** 1 Department of Traumatic Surgery, Shanghai East Hospital, School of Medicine, Tongji University, Shanghai 200092, China

**Keywords:** Sarcopenia, mendelian randomization, osteoporosis, IL-33, myokines, osteokines

## Abstract

**Introduction:**

Inflammaging, a term describing the chronic, low-grade inflammatory state associated with aging, is implicated in various age-related diseases. Inflammatory proteins are central to inflammaging and bone-muscle communication. This study sought to investigate the role of inflammatory proteins as potential myokines and osteokines in the context of sarcopenia-osteoporosis crosstalk using a convenient and efficient method, Mendelian Randomization (MR).

**Methods:**

We employed a network-based MR approach, structuring analyses into forward and reverse directions. In forward analyses (inflammatory proteins as exposures), we examined their impact on sarcopenia traits and osteoporosis. Reverse analyses (inflammatory proteins as outcomes) assessed the influence of sarcopenia and osteoporosis on protein levels. Our analysis included 91 inflammatory proteins, three sarcopenia traits, and osteoporosis outcomes.

**Results:**

In forward analyses, we identified 24 significant causal relationships between inflammatory proteins and outcomes. Reverse analyses revealed 40 significant associations. Seven proteins emerged as key mediators of sarcopenia-osteoporosis interactions: IL-33, RANKL, CXCL1, CXCL5, CX3CL1, TNFB, and CD5. Notably, a causal link was found between poor handgrip strength and downregulated IL-33 (effect = -0.104), and downregulated IL-33 was causally associated with an increased risk of osteoporosis (effect = -0.003).

**Discussion:**

The identification of IL-33 as a potential novel myokine not only underscores the importance of inflammaging in musculoskeletal health but also highlights IL-33 as a promising therapeutic target for interventions targeting muscle-bone crosstalk in older adults. However, further experimental studies are warranted to validate the role of IL-33 in this context and to explore the underlying biological mechanisms.

**Conclusion:**

Our findings highlight IL-33 as a promising novel myokine involved in sarcopenia-osteoporosis communication. This MR analysis provides mechanistic insights into the inflammatory basis of these age-related conditions and suggests IL-33 as a potential therapeutic target for interventions aiming to improve muscle-bone health in older adults.

## 1 INTRODUCTION

Skeletal muscles and bones are two major components of the musculoskeletal system, and the degeneration of these two organs may contribute to the occurrence of sarcopenia and osteoporosis [1-3]. Sarcopenia, as well as osteoporosis, is significantly associated with poor outcomes in older adults, including fractures, disability, and mortality [4]. Both sarcopenia and osteoporosis are becoming more prevalent with aging, and many individuals may be diagnosed with both sarcopenia and osteoporosis; this status was defined as “osteosarcopenia” or “sarco-osteoporosis [5-7]. The prevalence of sarco-osteoporosis may reach 18.5%, and the prevalence varies in populations with different risk factors, including sex, regions, frailty, age, malnutrition, and so on [8].

Though the correlation between sarcopenia and osteoporosis was clear based on clinical studies with large sample sizes [9, 10], more and more evidence suggested their causal relationships: sarcopenia may contribute to the development of osteoporosis, and osteoporosis may also aggravate the sarcopenia status [11, 12]. This interaction may be based on multiple factors, such as biomechanics [13], physical exercises [14], endocrine [15], nutrition [16], inflammation [17], gene expression [18], and so on. In recent years, more and more studies have reported that myokines and osteokines may also play significant roles in bone-muscle crosstalk [12, 19-21]. Myokines and osteokines are the muscle- and bone-derived factors [22]. In addition to autocrine and paracrine, skeletal muscles and bones may secrete these factors into the circulation to exert their effects on multiple organs [23-25]. The currently identified myokines and osteokines include IL-6, IL-10, myostatin, irisin, RANKL, osteocalcin, and so on [26-28].

Sarcopenia and osteoporosis, as two age-related diseases, may also be regulated by the aging status [4, 12, 19]. Recent studies suggested that inflammaging, a chronic low-grade inflammation status associated with aging and characterized by elevated levels of pro-inflammatory factors and immune dysregulation, may have critical roles in both sarcopenia and osteoporosis [29-31]. Inflammatory proteins, as important variables in the development of inflammaging, may be the bridge to explore the relationships between inflammaging and sarcopenia, as well as osteoporosis [17].

Since most of the identified myokines and osteokines are also inflammatory proteins, identifying the inflammatory proteins as myokines and osteokines may provide us with potential strategies for both sarcopenia and osteoporosis and more understanding of inflammaging in sarcopenia-osteoporosis communications. By targeting the signals related to inflammatory proteins, we may develop novel and satisfactory drugs to simultaneously enhance the quality and mass of both muscle and bone in older adults and to inhibit the development of sarcopenia and osteoporosis. However, despite the identification of several meaningful myokines and osteokines in previous studies [12, 19], there is a need to explore more potential factors and understand their roles in bone-muscle interaction. Identifying novel myokines and osteokines could provide more comprehensive insights into sarcopenia-osteoporosis communication and inform the development of therapeutic strategies.

Usually, identifying a novel myokine or osteokine requires a large amount of clinical data and basic experience [32]. In recent years, with the development of genetics, we may explore the potent myokines and osteokines using a novel genetic method, Mendelian Randomization (MR). MR is a methodological approach using genetic variants as instrumental variables (IVs) to investigate causal relationships between exposures and outcomes [33-35]. IVs are variables that are correlated with the exposure but not directly with the outcome, except through their effects on the exposure. In this study, we aim to address this research gap by using MR, a genetic epidemiological method that leverages genetic variants as instrumental variables to investigate causal relationships between exposures and outcomes. MR is performed based on genome-wide association studies (GWASs), and single-nucleotide polymorphisms (SNPs) are used as IVs. MR may infer causal associations between circulating inflammatory proteins and sarcopenia-osteoporosis communication with lower cost and higher levels of evidence, as it minimizes the impact of confounding variables and reverse causation, common challenges in observational studies of complex biological systems like bone-muscle crosstalk. By using the bidirectional MR method, we may estimate the factors that are causally associated with sarcopenia or osteoporosis, and by integrating the results of forward and reverse analyses, we may map a causal network of sarcopenia, osteoporosis, and inflammatory proteins, which may efficiently predict the potential myokines and osteokines in muscle-bone crosstalk.

## 2 MATERIALS AND METHODS

### 2.1 Study Design

This study was based on bidirectional two-sample Mendelian Randomization studies. Our study was conducted by using publicly accessible GWAS summary data obtained on November 20, 2023. Given the characteristics of GWAS summary data, no personally identifiable information of individual participants was retrieved at any point during or after data collection. There was no necessity for additional ethics approval or consent to participate. Details regarding ethics approval and consent to participate in each GWAS were available in the original studies.

In brief, to map the network of causal relationships between inflammatory proteins, sarcopenia, and osteoporosis, we performed eight groups of MR analysis. In Analysis groups A to D (forward analysis), we set 91 inflammation-related proteins as exposures and three sarcopenia traits (hand grip strength, adjusted appendicular lean mass, and usual walking pace), as well as osteoporosis, as outcomes. This approach allows us to investigate the potential causal effects of inflammatory proteins on sarcopenia and osteoporosis. In Analysis groups E to H (reverse analysis), we conducted reverse MR analyses where the 91 inflammation-related proteins were designated as outcomes, while the three sarcopenia traits and osteoporosis served as exposures. The reverse MR analyses may investigate the possibility of reverse causation, specifically whether sarcopenia and osteoporosis could influence the levels of inflammatory proteins. This bidirectional approach allows for a more thorough exploration of the relationship between these variables, offering deeper insights into how inflammatory proteins and sarcopenia/osteoporosis traits might interact with each other.

The results of the MR analyses were further reviewed to establish the network. By interpreting the network, the potential feedback factors, myokines, and osteokines were identified. The overall design of this study is shown in Fig. (**1**).

### 2.2 Cohorts and Traits

The summary GWAS data on 91 circulating inflammatory proteins were obtained from a meta-analysis including 14,824 individuals of European descent from 11 cohorts [36]. Inclusion and exclusion criteria were applied at the GWAS level rather than to individual participants. The inclusion criteria were: a. similar ancestry (to minimize population stratification); b. publicly released GWAS summary statistics; c. sample size ≥ 5,000; d. availability of full summary statistics, including effect alleles, non-effect alleles, effect estimates, standard errors, and allele frequencies. The exclusion criteria were: a. GWAS with overlapping cohorts; b. studies restricted to pediatric populations; c. studies restricted to specific disease subgroups. The detailed information on these proteins, including database ID, annotation, abbreviation, and sample size, is given in Supplementary Table **1**. Detailed information on these 11 cohorts is provided in Supplementary Table **2**. The proteins were measured using the Olink Target-96 Inflammation immunoassay panel.

Based on the principles of sarcopenia trait selection [37], three kinds of sarcopenia traits were selected: hand grip strength, adjusted appendicular lean mass (AALM), and usual walking pace. The summary GWAS for hand grip strength was performed on 254,894 older adults from 22 cohorts [38], and the poor hand grip strength was assigned as the case according to the EWGSOP definition (male: < 30 kg; female: < 20kg). The cohorts of AALM were from 450,243 populations in the UK Biobank cohort, and the AALM scores were measured by the BIA approach [39]. The data on the usual walking pace were collected based on 459,915 participants in the UK Biobank cohort, and the trait was defined as a categorical ordered variable (c, steady, and slow). The summary GWAS data of osteoporosis were obtained from the UK Biobank, and this cohort included 462,933 individuals. The detailed information on all cohorts related to the traits that were included in our study is summarized in Supplementary Table **2**. The cohorts of inflammatory factors and the cohorts of sarcopenia and osteoporosis were independent of each other, which satisfied the basic assumption of two-sample MR analysis.

### 2.3 Mendelian Randomization Analyses

All the significance thresholds for selecting the IVs that were associated with exposures in this study were set as *p* < 5 × 10^-6^ to obtain an adequate number of IVs. To meet the three basic assumptions for MR analysis and to reduce the bias caused by week IVs and horizontal pleiotropy, we conducted six strict IV filtration steps following previous protocols [33]. Firstly, to ensure that the IVs were independent of each other and to avoid linkage disequilibrium, we conducted a clumping process. This involved setting a threshold of r^2^ < 0.01 within a 10 kb window. Secondly, to guarantee the specificity of the IVs to the exposures of interest, we excluded SNPs associated with more than one exposure. Thirdly, to satisfy the independence assumption, we eliminated SNPs associated with potential confounders such as age, body mass index (BMI), sex, whole-body impedance, and weight. Fourthly, during the data harmonization process, palindromic SNPs were removed to ensure the accuracy and consistency of the data. Fifthly, to satisfy the exclusion restriction assumption, we excluded SNPs that were associated with the outcomes at a threshold of *p* < 5 × 10^−5^. Lastly, to avoid the potential bias caused by horizontal pleiotropy, a situation where a genetic variant affects the outcome through pathways unrelated to the exposure, the MR-PRESSO outlier test was applied to detect and remove outlier SNPs.

Then, eight groups of MR analyses were conducted according to the design of analysis groups A-H mentioned above. Random-effects inverse-variance weighted (RE IVW) analysis was employed as the main method to explore the causal relationships due to its much more conservative estimate ability. To assess the strength of IVs and evaluate the stability of the results of the main MR analyses, multiple further analyses were performed as sensitivity analyses. Firstly, to detect the potential weak instruments, we calculated the F-statistics and R^2^ of each IV following the previous formula. The IV with an F statistic lower than 10 was defined as a weak instrument. Next, to further prove the results of RE IVW, multiple MR models were established, including MR-Egger regression, weighted median (WME), weighted mode (WMD), simple median (SM), fixed effects inverse-variance weighted (FE IVW), and robust inverse-variance weighted (RO IVW). Finally, the MR-Egger intercept test was used to assess the directional pleiotropy, and Cochran’s Q test was performed to detect heterogeneity.

### 2.4 Statistical and Network Analyses

In this study, all *p*-values < 0.05 were considered statistically significant. To map the network of inflammatory factors, sarcopenia, and osteoporosis, all the significant causal linkages, including forward analyses and reverse analyses, were collected. Then the raw network was reviewed, and those relationships that conflicted with each other were excluded. All the analyses were performed using R software version 4.2.2 (R Foundation for Statistical Computing, Vienna, Austria). MR and related sensitivity analyses were conducted by using R packages “TwoSampleMR” and “MendelianRandomization”. The results were visualized by using R packages “ComplexHeatmap”, “circlize”, and “RColorBrewer”.

## 3 RESULTS

### 3.1 General Information

The cohorts for circulation inflammatory factors included 14,824 individuals, and for sarcopenia traits, the sample sizes ranged from 254,894 to 459,915, and for the osteoporosis cohort, 462,933 individuals were included (Supplementary Table **2**). SNPs after filtration steps for inflammatory proteins were included in forward analyses, and SNPs for hand grip strength, AALM, usual walking pace, and osteoporosis were included in reverse analyses. Due to the insufficient number of SNPs for inflammatory proteins C-X-C motif chemokine 6 (CXCL6) and STAM binding protein (STAMPB), the estimates for the causal effects of these on osteoporosis were omitted. The detailed numbers of SNPs in each filtration step for all relationships are summarized in Supplementary Tables **3**-**4**. The detailed information on SNPs associated with multiple circulating inflammatory proteins in forward analyses (n = 121) and those associated with multiple sarcopenia traits in the reverse analysis (n = 2) is summarized in Supplementary Tables **5** and **6**, respectively. SNPs associated with confounders were detected and excluded in step 3 (Supplementary Table **7**). Finally, 1621 and 1195 SNPs were included in forward and reverse analyses, respectively (Supplementary Tables **8**-**9**).

### 3.2 Forward Analyses

Four groups of MR analyses were included as forward analyses: the 91 kinds of circulation inflammatory proteins were set as exposures, and hand grip strength, AALM, usual walking pace, and osteoporosis were set as four kinds of outcomes. The detailed results of these four groups of MR analyses are summarized in Supplementary Tables **10**-**13**. The overall results of RE IVW of these four groups of MR analyses were visualized in the four outer donuts, as shown in Fig. (**2**). In the analysis group A (inflammatory factors as exposures, hand grip strength as outcome), six factors were identified to causally relate to hand grip strength, including T-cell surface glycoprotein CD5 (CD5), Interleukin-6 (IL-6), and so on. Moreover, analysis group B (inflammatory factors as exposures, AALM as outcome) showed that 13 factors, including Fractalkine (CX3CL1), Fms-related tyrosine kinase 3 ligand (Flt3L), Interleukin-2 (IL-2), Tumor necrosis factor-beta (TNFB), TNF-related activation-induced cytokine (TRANCE), and so on, were causally associated with AALM. In the analysis group C (inflammatory factors as exposures, usual walking pace as outcome), the causal effects of eight inflammatory factors on usual walking pace were proven, such as CX3CL1 and Neurotrophin-3 (NT-3). Analysis group D was performed to explore the causal associations of inflammatory factors with osteoporosis, and in these analyses, more than 10 factors, including Interleukin-33 (IL-33) and TRANCE, were identified. Detailed results of these estimates are presented in Fig. (**3A**).

### 3.3 Reverse Analyses

Analysis groups E-H were defined as reverse analyses: 91 inflammation-related proteins were set as outcomes, three kinds of sarcopenia traits (hand grip strength, AALM, usual walking pace), and osteoporosis were set as exposures. The detailed results of these four groups of MR analyses are summarized in Supplementary Tables **14**-**17**. In reverse analyses, hand grip strength was proven to be causally related to IL-33. For AALM, its causal effects on 13 inflammatory factors were identified, including CD5, CX3CL1, C-X-C motif chemokine 1(CXCL1), C-X-C motif chemokine 5 (CXCL5), Delta and Notch-like epidermal growth factor-related receptor (DNER), Interleukin-10 receptor subunit beta (IL-10RB), Interleukin-24 (IL-24), Signaling lymphocytic activation molecule (SLAMF1), TNFB, and Tumor necrosis factor receptor superfamily member 9 (TNFRSF9). Moreover, the causal relationships between the usual walking pace and eight factors were proven, including CXCL1, CXCL5, DNER, and so on. The causal associations of osteoporosis with NT-3 and TNFB were also detected. The overall results of RE IVW of these four groups of MR analyses were visualized in the four inner donuts, as shown in Fig. (**2**). Detailed results of significant estimates are provided in Fig. (**3B**).

### 3.4 Sensitivity Analyses

In sensitivity analyses for forward analysis (Fig. **4**), the majority of the causal estimates were not proven to have directional pleiotropy and heterogeneity, except for the analyses for the causal associations of IL2, Flt3L, and TNFB with AALM.

In sensitivity analyses for reverse analysis (Fig. **4**), most of the causal estimates were not proven to have directional pleiotropy and heterogeneity. However, heterogeneity was detected in the causal associations of AALM and six factors, including CCL23, CD5, CSF-1, CX3CL1, CXCL5, IL-10RB, and TNFRSF9, and directional pleiotropy was identified in the causality between AALM and SLAMF1 (all *p* < 0.05).

### 3.5 Network Analyses

All the significant associations identified in forward and reverse analyses are summarized in Supplementary Table **18**. Integrating the significant causal linkages, we mapped the causal network between sarcopenia, osteoporosis, and circulation inflammatory proteins. In this network, the traits of sarcopenia, osteoporosis, and proteins were presented by the points, and the lines meant the significant causalities (Fig. **5A**). However, in the raw network, several pairs of causal associations were mutually contradictory. For example, AALM may have a causally positive effect on DNER (effect = 0.091), while the usual walking pace’s causal effect was negative (effect = -0.015). Therefore, we further reviewed the raw network and omitted the associations that were difficult to interpret.

As shown in Fig. (**5B**), seven proteins were finally included in the network. By interpreting the map, we noted several interesting pathways. Firstly, CXCL5 and CXCL1 were proven to be the outcomes of AALM and usual walking pace: increased AALM and brisk usual walking pace may causally relate to the decreased serum levels of CXCL5 and CXCL1 (all effects < 0). Next, CD5 may play a mediating role in the communications between hand grip strength and AALM: the increase of AALM may causally relate to the upregulation of CD5 (effect = 0.077), and the high levels of CD5 were causally and negatively associated with poor hand grip strength (effect = -0.064). Moreover, CX3CL1 was proven to play its role between AALM and the usual walking pace. On the one hand, upregulated CX3CL1 was causally associated with low AALM and slow usual walking pace (all effects < 0), on the other hand, the network indicated that the CX3CL1 may be the negative feedback factors: low AALM may also downregulated the levels of CX3CL1 (effect = 0.091), which could potentially lead to a negative feedback loop. TNFB may play a positive feedback role: increased AALM was causally associated with TNFB positively, and the increase of TNFB was also causally related to the increased AALM (all effects > 0). TRANCE was identified as exposure to both AALM (effect = -0.022) and osteoporosis (effect = 0.002).

IL-33 may play a significant role in the communication between hand grip strength and osteoporosis: poor hand grip strength may be causally related to downregulated IL-33 (effect = -0.104), and downregulated IL-33 was causally associated with a high risk of osteoporosis (effect = -0.003). These findings suggest that low grip strength may promote the development of osteoporosis by downregulating IL-33, and IL-33 could be a potential myokine.

## 4 DISCUSSION

Sarcopenia and osteoporosis are two common diseases of the motor system in older adults, and exploring their communications may provide us with new insights into bone-muscle crosstalk and novel intervention targets for inhibiting the development of both sarcopenia and osteoporosis [32, 40-42]. Myokines and osteokines were proven to play roles in sarcopenia-osteoporosis communications, and most discovered myokines and osteokines were also inflammatory proteins [43, 44]. Based on these insights, identifying inflammatory proteins as novel cytokines or osteokines may not only expand our knowledge in bone-muscle cross-talk but also provide novel understandings of the relationships between inflammaging and sarcopenia, as well as osteoporosis. This might be a novel and systematic view into sarcopenia-osteoporosis communications.

In this study, the causal relationships between hand grip strength and IL-33 (effect = -0.104) and between IL-33 and osteoporosis (effect = -0.003) were significantly proven. Therefore, we speculate that the occurrence of sarcopenia, especially the decrease in grip strength, leads to a reduction in IL-33, thereby inhibiting the inhibitory effect of IL-33 on the occurrence and development of osteoporosis. Integrating the evidence from forward analyses and reverse analyses, we believe that IL-33 may be a novel myokine. IL-33 is a cytokine belonging to the IL-1 family, known for its crucial role in modulating immune responses and inflammation [45, 46]. Upon release, IL-33 binds to its receptor ST2, activating signaling pathways that regulate various immune cells, such as T helper cells and mast cells [47, 48]. This interaction plays a pivotal role in mediating allergic responses, tissue repair, and maintaining immune homeostasis [49, 50]. The relationships between IL-33 and muscle have been widely discussed in the recent decade, and firstly, the roles of IL-33 in the myocardium and smooth muscle attracted attention [51-53]. Gradually, the protective effects of IL-33 on skeletal muscles were also reported. IL-33 may promote muscle repair by regulating the accumulation of regulatory T cells (Tregs), and Tregs play key roles in inhibiting muscle atrophy [54, 55]. However, few studies explored whether muscle atrophy may cause a change in IL-33 level, and more experimental research is expected to be conducted to prove our estimation.

We proved that high levels of IL-33 may causally relate to the low risk of osteoporosis in this study by using MR. This view was also reported in previous studies, which suggested the stability and robustness of our results from MR. IL-33 inhibits bone loss *via* the IL-33/IL-31 axis: IL-33 regulates IL-31 secretion and inhibits the activity of osteoclasts by influencing RANKL [56]. Moreover, IL-33 may inhibit the formation and differentiation of osteoclasts [57]. In addition to osteoclasts, IL-33 may also contribute to osteogenic differentiation directly and regulate the inflammatory microenvironment, which promotes bone regeneration [58]. IL-33/ST2 was also a significant pathway involved in bone loss: ST2 may play its roles site-dependently and estrogen-dependently and regulate inflammatory bone destruction [59, 60].

Myokines are signaling molecules released by skeletal muscles that can regulate bone metabolism [22, 61, 62]. Given the causal effects observed between reduced grip strength and decreased IL-33 levels, as well as between lower IL-33 levels and increased osteoporosis risk, IL-33 shows potential as a myokine. By integrating the conclusion from previous studies and the results in our study, we estimated the potential of IL-33 as a myokine regulating osteoporosis. However, this interpretation remains speculative and requires further experimental validation. Future research could further explore IL-33's role as a myokine and osteokine through animal models, such as *IL-33* gene knockout or overexpression mice. These experiments would help clarify whether IL-33 can be released by skeletal muscles and its specific effects on bone metabolism. Additionally, *in vitro* studies on muscle cell lines or primary muscle cells could investigate the expression and release of IL-33 under different physiological and pathological conditions. More research is needed to clarify the exact mechanisms and roles of IL-33 in these processes.

As shown in Fig. (**5A**), the other three factors were also identified as potential mediating factors in sarcopenia-osteoporosis communications, including TNFB, IL-24, and NT3. However, the relationships between them and osteoporosis and sarcopenia were paradoxical. The effects mediated by them were inverse of each other, which is inconsistent with our premise that the relationship between sarcopenia and osteoporosis is mutually causal. Though previous studies have reported the roles of TNFB, IL-24, and NT3 in sarcopenia and osteoporosis [63-66], more studies were needed to confirm whether they can act as myokines. Besides the mediating factors in sarcopenia-osteoporosis, TRANCE was found to be a cause for both sarcopenia and osteoporosis. TRANCE, also known as RANKL, is a key cytokine involved in regulating bone metabolism and immune responses [67]. RANKL is a star factor in muscle and bone metabolism, and its role in osteoporosis and sarcopenia has been proven strongly [68-70]. In clinical practice, denosumab, an inhibitor of RANKL, has been developed as a novel targeted therapy for osteoporosis.

In this study, in addition to the factors playing roles in sarcopenia-osteoporosis communications, some factors’ regulative roles in sarcopenia traits were also explored. CXCL1 and CXCL5 were found to be the outcomes of both AALM and usual walking pace. CD5 was proven to be the mediating factor between AALM and hand grip strength. TNFB might be a positive feedback factor in AALM. CX3CL1 may be a protective factor for both AALM and the usual walking pace, and may be a negative feedback factor for AALM. CXCL1 was used in many studies as an indicator of inflammatory status in skeletal muscle and was identified as a myokine [71]. When the muscle atrophy was alleviated, the reduced levels of CXCL1 were also found, which indicated that CXCL1 might be the outcome of changes in muscle metabolism [72, 73]. Though few studies reported the relationships between CXCL5 and sarcopenia, CXCL5, as a chemokine, may play a similar role as CXCL1. The role of CX3CL1 in sarcopenia was further proven in our study: CX3CL1 was identified as a myokine in previous studies [74, 75]. CX3CL1 may exert its effects on muscle by regulating neutrophil recruitment [71].

We have to admit that some identified myokines and osteokines have no significant effects in our study, such as IL-6, IL-1, and so on [28, 76]. The reason why the causal links between sarcopenia and osteoporosis were not significantly strengthened may be that these factors may exert their effects *via* paracrine. We used the GWASs of circulating inflammatory factors, so some identified myokines and osteokines that may play roles in local tissues may not be significant in this study.

However, comparing our findings with existing knowledge, many factors indeed have been confirmed to play roles in the regulation of skeletal muscle and bone, such as RANKL, TNFB, CXCL1, and so on. This further underscores the effectiveness and robustness of our use of the MR model to explore circulating inflammatory factors in skeletal muscle and bone. By employing this model, it is possible to infer factors related to muscle and bone metabolism, as well as myokines and osteokines. Therefore, in this study, the identification of a new muscle factor, IL-33, although its role as a myokine has been less studied, based on the predictive efficacy of this study on other factors, IL-33 has the potential to act as a muscle factor. Furthermore, this study pioneered a novel approach, utilizing Mendelian randomization models and genetic evidence, to investigate disease-related molecular mechanisms.

This study had some limitations. Firstly, the generalizability of our findings to non-European populations is uncertain. The majority of the genetic data utilized in our study were derived from European populations, which may limit the applicability of our results to other ethnic groups. Future studies should strive to incorporate larger and more diverse cohorts to enhance the generalizability of the results. Secondly, evidence of directional pleiotropy was observed in some causal effect assessments. This may introduce bias and affect the validity of the results. However, since these causal relationships were not included in the network analysis and were not further discussed, their potential impact on our study conclusions is likely minimal. Thirdly, while some MR analyses in our study exhibited heterogeneity, the random-effects IVW model, known for its conservative nature, might mitigate the impact of heterogeneity. Moreover, this study aimed to establish a novel method to detect the potential myokines and osteokines, so further experimental studies should be conducted to prove our predictions. Additionally, given that our study relied on summary-level data from these GWAS, the sample size was not calculated de novo for our specific MR analyses. Lastly, we used circulating inflammatory proteins as potential factors involved in sarcopenia and osteoporosis, while some proteins that act as myokines or osteokines and play their roles in local tissues may not be detected in this study.

## CONCLUSION

This study systematically explored the causal roles of inflammatory proteins in sarcopenia-osteoporosis communication using bidirectional Mendelian randomization, identifying IL-33 as a potential novel myokine that may regulate bone-muscle crosstalk. Key findings revealed that reduced hand grip strength is causally associated with lower IL-33 levels, which in turn are linked to an increased risk of osteoporosis, suggesting a possible mechanistic pathway connecting sarcopenia to bone deterioration. The integration of genetic evidence and network analysis provided a robust framework for prioritizing inflammatory proteins as potential myokines or osteokines, advancing our understanding of inflammaging in age-related musculoskeletal decline. Notably, this study pioneers the application of MR to map causal networks in bone-muscle crosstalk, offering a scalable method for identifying potential therapeutic targets.

However, our findings must be interpreted with caution. The role of IL-33 as a novel myokine is suggested by the data but requires further experimental validation. The therapeutic potential of IL-33 for sarcopenia and osteoporosis remains hypothetical and should be framed as such. Limitations such as population specificity and residual pleiotropy in some analyses may influence the study’s conclusions and should be addressed in future research. Future studies should validate these results in diverse cohorts, elucidate IL-33’s tissue-specific mechanisms through experimental models, and explore synergistic effects of targeting IL-33 with existing therapies. Ultimately, while this work bridges genetic insights with clinical translation, further experimental evidence is needed to confirm IL-33’s role as a myokine and its therapeutic potential in mitigating osteosarcopenia.

## Figures and Tables

**Fig. (1):**
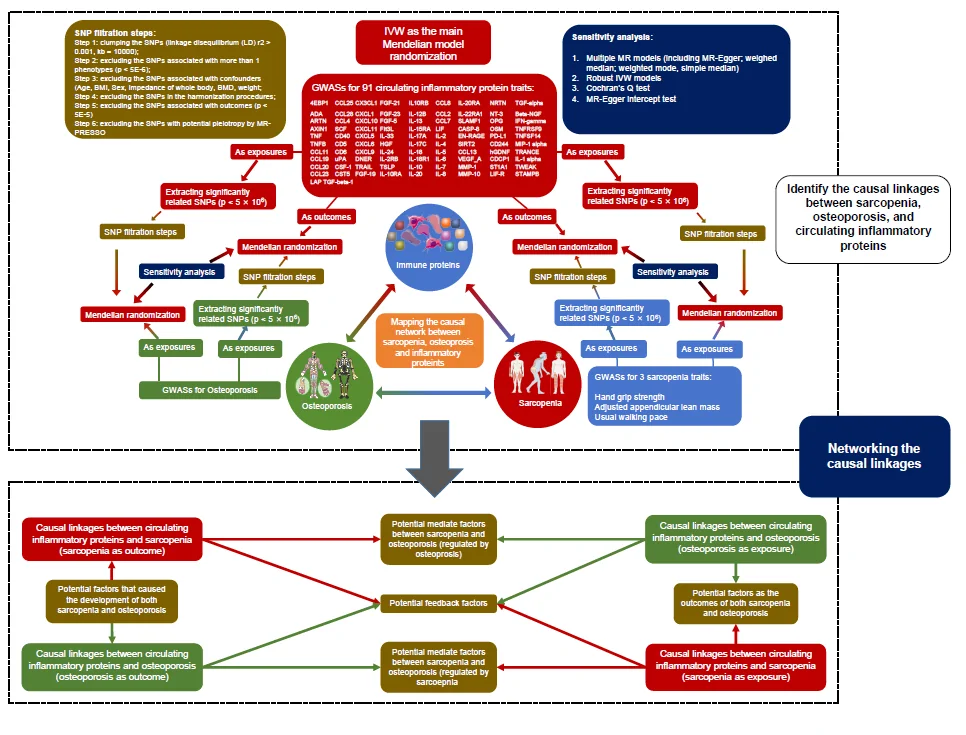
Overall design of our study.

**Fig. (2):**
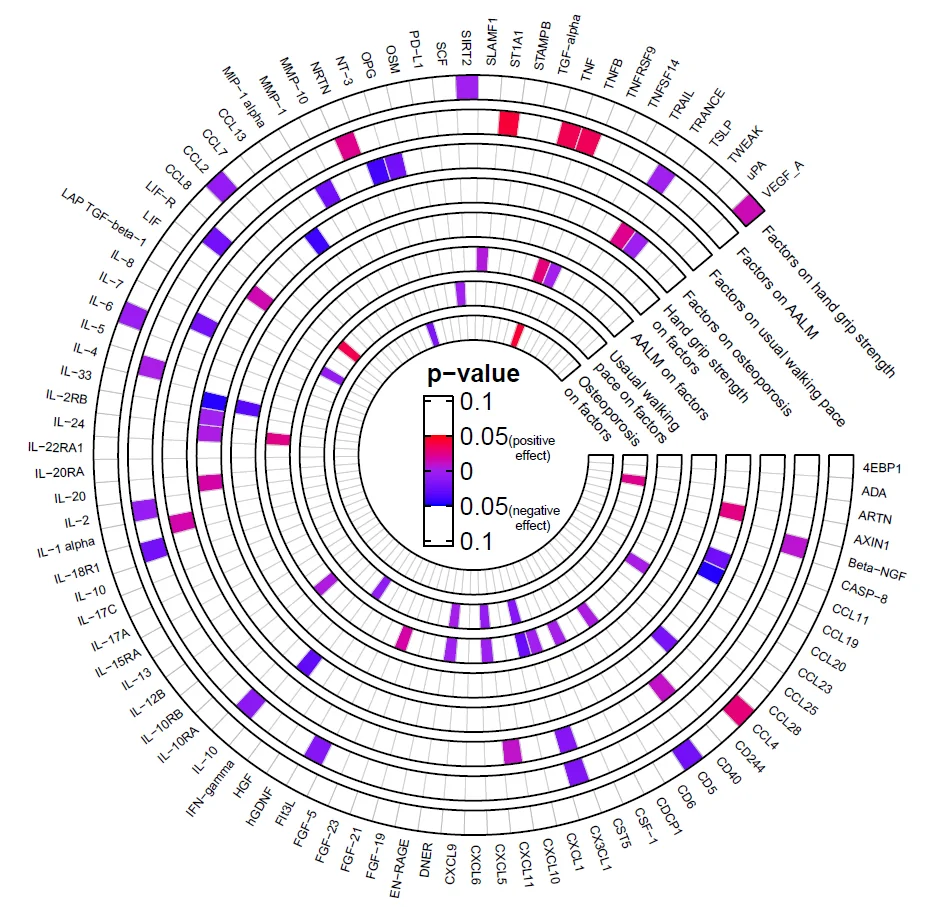
Overall results of RE IVW in this study (*p* values). The results of analyses A-H are represented by the eight concentric rings from outer to inner.

**Fig. (3):**
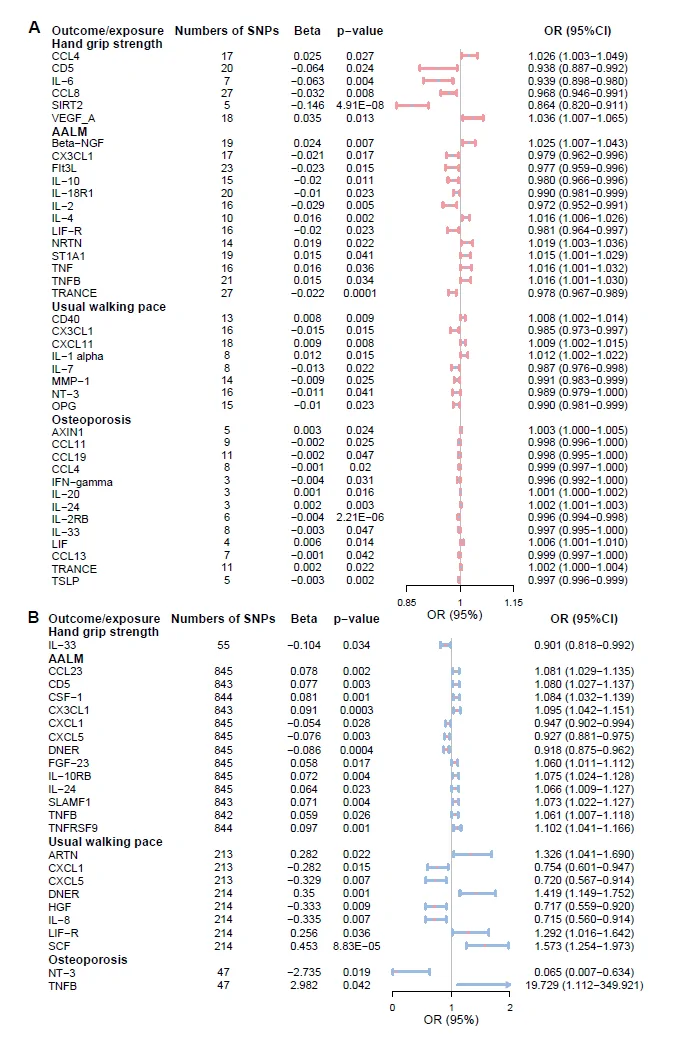
Detailed information on causal relationships with statistical significance. (**A**) Forward analysis; (**B**) Reverse analysis.

**Fig. (4):**
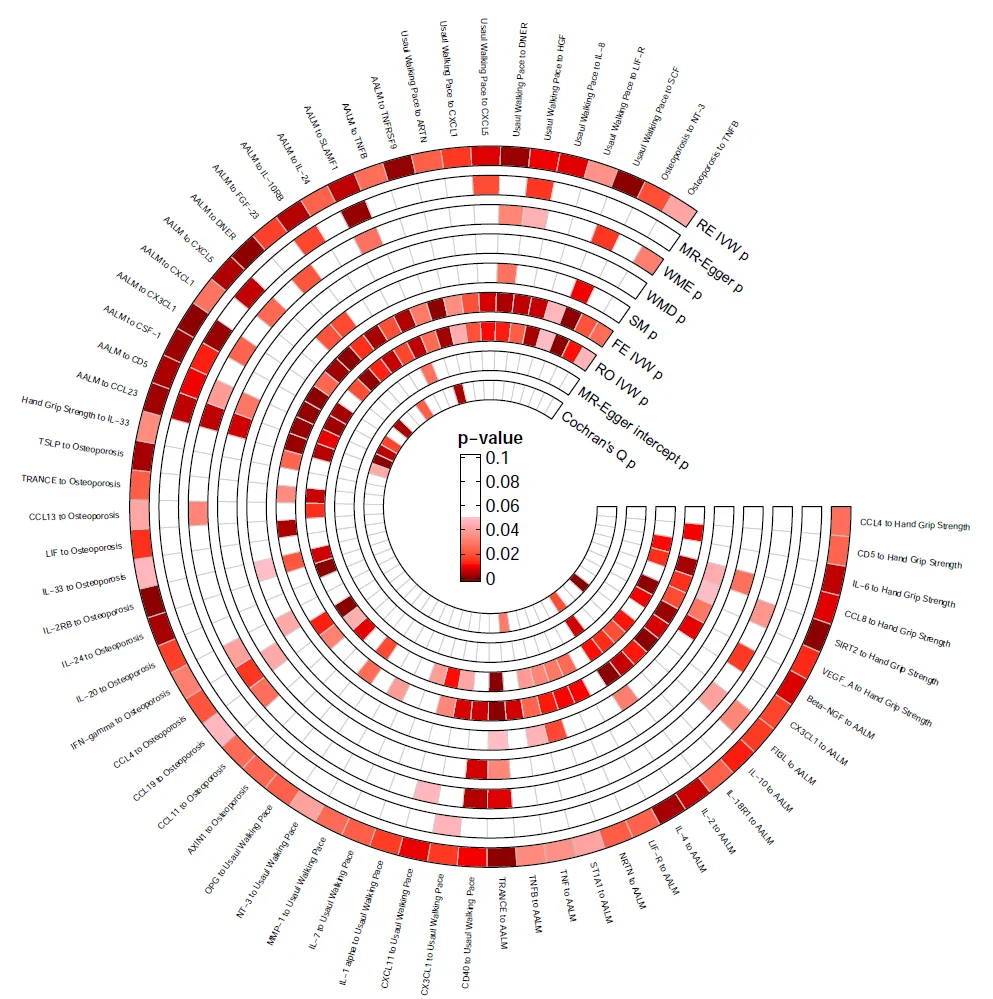
Overall results of sensitivity analyses for causal relationships with statistical significance (*p* values). The results of different sensitivity analyses are represented by the concentric rings from outer to inner. From outer to inner: Random-effects inverse-variance weighted (RE IVW), MR-Egger, weighted median (WME), weighted mode (WMD), simple median (SM), fixed effects IVW (FE IVW), and robust IVW (RO IVW).

**Fig. (5):**
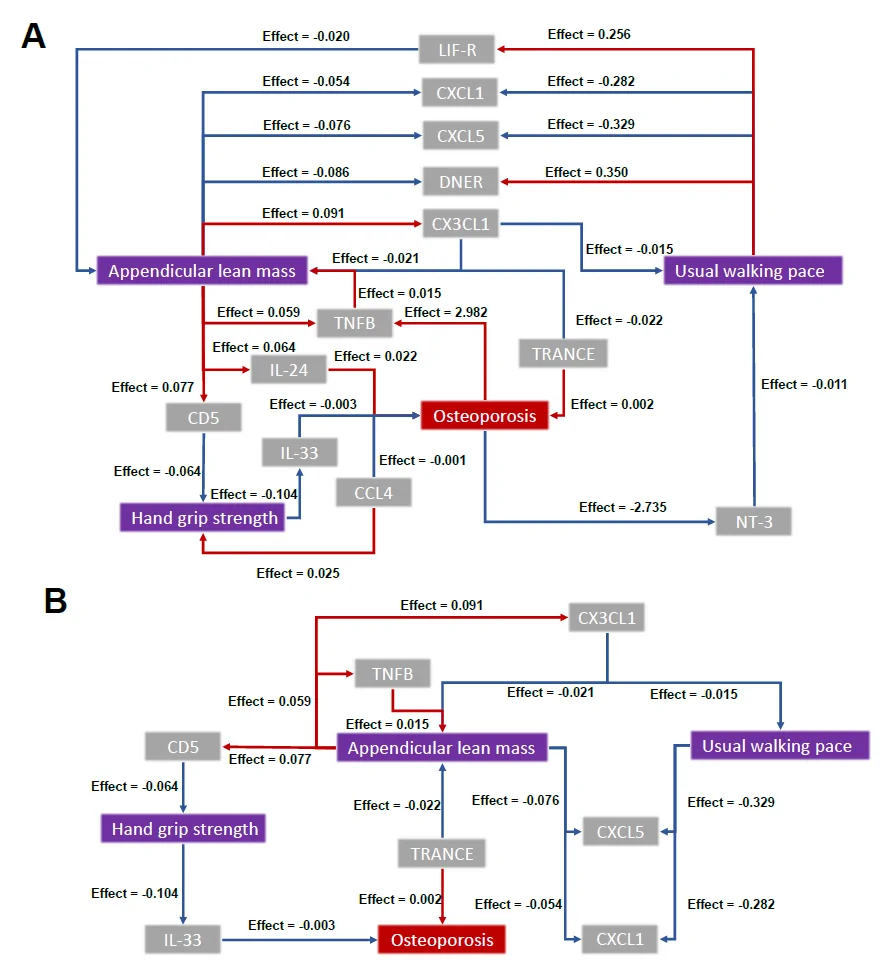
Causal network between sarcopenia, osteoporosis, and inflammatory factors. The traits of sarcopenia, osteoporosis, and proteins were presented in the points. Lines with arrows represent significant causal relationships, with exposure pointing towards the outcome. The color of the lines indicates the effect, with positive effects shown in red and negative effects in blue. A: Raw network; B: Network after omitting the mutually contradictory associations.

## Data Availability

All data generated or analyzed during this study are included in this published article.

## LIST OF ABBREVIATIONS

AALM: Adjusted Appendicular Lean Mass
CD5: Cluster of Differentiation 5
CX3CL1: C-X3-C Motif Chemokine Ligand 1
CXCL1: C-X-C Motif Chemokine Ligand 1
CXCL5: C-X-C Motif Chemokine Ligand 5
CXCL5: C-X-C Motif Chemokine Ligand 6
CCL23: Chemokine (C-C Motif) Ligand 23
CSF-1: Colony Stimulating Factor 1
DNER: Delta and Notch-like Epidermal Growth Factor-related Receptor
FE IVW: Fixed-effects Inverse-variance Weighted
Flt3L: Fms-Related Tyrosine Kinase 3 Ligand
GWAS: Genome-wide Association Studies
IL-10: Interleukin-10
IL-2: Interleukin-2
IL-24: Interleukin-24
IL-33: Interleukin-33
IL-6: Interleukin-6
IL-10RB: Interleukin-10 Receptor Subunit Beta
IVs: Instrumental Variables
MR: Mendelian Randomization
NT-3: Neurotrophin-3
RE IVW: Random-effects Inverse-variance Weighted
RANKL: Receptor Activator of Nuclear Factor-kB Ligand
RO IVW: Robust Inverse-variance Weighted
SLAMF1: Signaling Lymphocytic Activation Molecule Family Member 1
SM: Simple Median
SNPs: Single-nucleotide Polymorphisms
STAMPB: STAM Binding Protein.
TNFB: Tumor Necrosis Factor-beta
TNFRSF9: Tumor Necrosis Factor Receptor Superfamily Member 9
TRANCE: TNF-Related Activation-induced Cytokine
WME: Weighted Median
WMD: Weighted Mode
